# MiR‐223‐3p plays a role in sex‐biased intercellular communication between macrophages and microglia

**DOI:** 10.1002/alz.090000

**Published:** 2025-01-03

**Authors:** Paresh Prajapati, Urim Geleta, Wang‐Xia Wang

**Affiliations:** ^1^ University of Kentucky/Sanders‐Brown Center on Aging, Lexington, KY USA

## Abstract

**Background:**

The characterization of intercellular communication between peripheral immune cells and the central nervous system (CNS) are essential for understanding the brain’s response to aging and disease states, such as Alzheimer’s disease. MicroRNAs (miRNAs) constitute a class of small non‐coding RNAs that play a crucial role in regulating various biological and pathological processes, including those related to immunity and inflammation. MiR‐223‐3p, residing on the X chromosome, is a pivotal miRNA involved in the inflammatory response, with its expression being enriched in macrophages/microglia. However, the impact of miR‐223‐3p on the intercellular communication and the subsequent neuroinflammation remains unclear.

**Method:**

MiR‐223‐3p knockout (KO) and genetic background matched wildtype (WT), female and male mice were used in the studies. Microglia were isolated from the brain tissues of 3‐week‐old pups and enriched using the EasySep™ Mouse CD11b Positive Selection Kit. Bone marrow‐derived macrophages (BMDMs) were isolated from 6 months old mice. Microglia isolated from WT mice were co‐cultured with BMDMs isolated from 1) female miR‐223‐3p KO, 2) male miR‐223‐3p KO, 3) female WT, and 4) male WT mice. The BMDMs were pre‐treated with LPS/IFNγ or vehicle control media before incubating with microglia for 24 hrs. RNAs were then extracted from microglia for RT‐qPCR quantification of inflammatory markers.

**Result:**

Our initial screening of gene expression through RNA‐seq studies reveals that the absence of miR‐223‐3p in bone BMDMs leads to a notable sex‐specific alteration in the expression of immune and inflammatory genes. RT‐qPCR analysis of microglia revealed a distinct alteration in the expression of inflammatory genes when co‐cultured with KO BMDMs relative to WT BMDMs. In particular, microglia co‐cultured with BMDMs from KO mice induced a substantial expression of *IL1b, TNFα, CXCL10, H2Aa, H2EB1*, and *APOE* compared to WT mice. Nevertheless, strong induction of *CD86* was observed in microglia co‐cultured with male KO BMDMs compared to that of female KO BMDMs, while *APOE, H2Ab1*, and *CD74* were higher in microglia co‐cultured with female KO BMDMs.

**Conclusion:**

These preliminary results suggest that the expression of miR‐223‐3p in peripheral macrophages may play an important role in sex‐biased inter‐cellular communication that ultimately will impact the inflammatory response in the CNS.

